# Generation of Soluble Interleukin-11 and Interleukin-6 Receptors: A Crucial Function for Proteases during Inflammation

**DOI:** 10.1155/2016/1785021

**Published:** 2016-07-14

**Authors:** Juliane Lokau, Maria Agthe, Christoph Garbers

**Affiliations:** Institute of Biochemistry, Kiel University, 24118 Kiel, Germany

## Abstract

The cytokines interleukin-11 (IL-11) and IL-6 are important proteins with well-defined pro- and anti-inflammatory functions. They activate intracellular signaling cascades through a homodimer of the ubiquitously expressed signal-transducing *β*-receptor glycoprotein 130 (gp130). Specificity is gained through the cell- and tissue-specific expression of the nonsignaling IL-11 and IL-6 *α*-receptors (IL-11R and IL-6R), which determine the responsiveness of the cell to these two cytokines. IL-6 is a rare example, where its soluble receptor (sIL-6R) has agonistic properties, so that the IL-6/sIL-6R complex is able to activate cells that are usually not responsive to IL-6 alone (trans-signaling). Recent evidence suggests that IL-11 can signal via a similar trans-signaling mechanism. In this review, we highlight similarities and differences in the functions of IL-11 and IL-6. We summarize current knowledge about the generation of the sIL-6R and sIL-11R by different proteases and discuss possible roles during inflammatory processes. Finally, we focus on the selective and/or combined inhibition of IL-6 and IL-11 signaling and how this might translate into the clinics.

## 1. Introduction

Interleukin-6 (IL-6) has often been regarded as a prototypical proinflammatory cytokine, and the development and clinical use of a humanized antibody against the IL-6 receptor (IL-6R) to treat inflammatory diseases further strengthens this notion [1, 2]. However, IL-6 can signal via membrane-bound (called classic signaling) and soluble forms of the IL-6R (sIL-6R, trans-signaling), and it has become clear that especially IL-6 trans-signaling accounts for the proinflammatory properties of the cytokine [3–5]. Specific inhibition of this pathway is sufficient and sometimes even superior compared to the total blockade of IL-6 [6].

In contrast, IL-11 is less well investigated and characterized in terms of its pro- and anti-inflammatory properties. Recombinant IL-11 (oprelvekin) is approved for the prevention of severe thrombocytopenia following chemotherapy and was always considered as acting rather as anti-inflammatory [7]. However, growing evidence suggests that IL-11 drives tumorigenesis within the stomach and the intestine independently of IL-6 [8, 9], which clearly indicates that IL-11 has to be considered as an important proinflammatory cytokine [7, 10–12]. Signaling of IL-11 is believed to solely occur via a membrane-bound IL-11R, but we have recently shown that also IL-11 can signal via a soluble IL-11R (sIL-11R) [13]. Whether this IL-11 trans-signaling pathway is also of special importance like the IL-6 trans-signaling pathway is not explored yet.

In this review, we describe signal transduction by IL-11 and IL-6 and summarize the current knowledge about the proteolytic cleavage of IL-6R and IL-11R, which leads to the generation of soluble agonistic cytokine receptors. Furthermore, we highlight the role of circulating soluble cytokine receptors in human blood and discuss therapeutic strategies to inhibit single or multiple IL-6 and IL-11 signaling pathways.

## 2. Classic Signaling and Trans-Signaling: Two Modes of Action for IL-11 and IL-6

**Figure 1:**
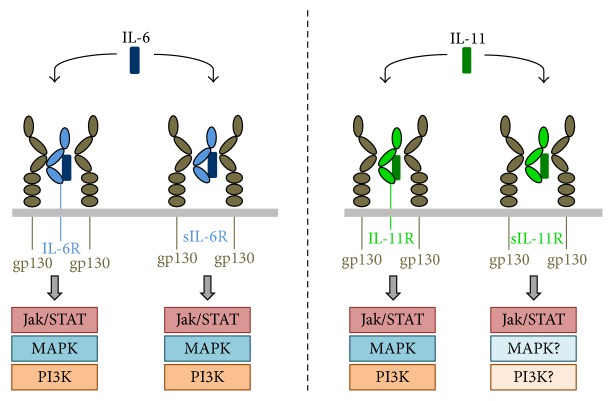
Schematic illustration of IL-6 and IL-11 signaling. IL-6 (dark blue) binds to membrane-bound and soluble forms of the IL-6R (light blue), which leads to gp130 (brown) homodimerization and subsequent activation of the intracellular signaling pathways Jak/STAT, MAPK, and PI3K. Similarly, IL-11 (dark green) can bind to both membrane-bound and soluble forms of the IL-11R (light green), and both lead to homodimerization of gp130. Whereas activation of Jak/STAT, MAPK, and PI3K has been shown for signaling via the membrane-bound IL-11R, formally only activation of the Jak/STAT pathway via the sIL-11R has been demonstrated.

The signal transduction of IL-6 and IL-11 is induced by binding of the cytokines to their specific nonsignaling *α*-receptors, IL-6R or IL-11R. The so formed complex then recruits two molecules of the signal-transducing *β*-receptor gp130, which dimerize and activate intracellular signaling molecules that result in activation of the Jak/STAT, PI3K, and MAPK pathways [14] (Figure 1). Notably, while both cytokines signal via the gp130 homodimer, and also the signaling pathways are similar, they are described to have distinct and, in part, opposing roles [7]. The *α*-receptors share the same topological organization. Their extracellular parts consist of an Ig-like domain (D1), which is followed by two fibronectin-type-III domains (D2 and D3) and a so-called stalk region. Binding of the respective cytokine is achieved by the cytokine-binding module (CBM), which is composed of domains D2 and D3. It has been shown that the stalk of the IL-6R is required to position the CBM in a certain distance from the membrane in order to allow efficient signal transduction [15]. The role of the IL-11R stalk has not been analyzed in this regard.

The signal transduction of IL-6 and IL-11 is solely mediated via the gp130 homodimer and does not directly involve the *α*-receptors [16, 17]. As the transmembrane and intracellular part of the *α*-receptors are not required for formation of the signaling complex [18, 19], IL-6 and IL-11 can also initiate dimerization of two gp130 molecules via their respective soluble *α*-receptor. Soluble IL-6 receptor (sIL-6R) is found in the serum of healthy humans in concentrations ranging from 30 to 70 ng/mL [20, 21] and has been shown to be moderately elevated under pathophysiological conditions [22, 23]. The serum levels of sIL-11R are remarkably lower, ranging from 20 pg/mL to 4 ng/mL. Interestingly, in contrast to sIL-6R, sIL-11R has been detected in the serum of some, but not all, healthy humans [13].

In contrast to other cytokines like IL-1*α* or TNF*α*, where the soluble receptors act as antagonists [24], sIL-6R and sIL-11R have been shown to mediate the signal transduction of their ligands [13, 17, 24–26]. Signaling via a membrane-bound receptor is termed classic signaling while signal transduction via soluble receptors is referred to as trans-signaling [24, 25]. Contradicting data exist on whether or not classic IL-11 signaling can be antagonized by sIL-11R [27, 28]. No antagonistic effect of sIL-6R has been described thus far.

IL-6 binds to its soluble *α*-receptors with the same affinity as to the membrane-bound forms [29]. This is necessary, because neither the cytokine nor the *α*-receptor alone can bind to gp130. It is still under debate whether the signal transduction of IL-6 is mediated via a tetrameric (IL-6/IL-6R/gp130_2_) or a hexameric (IL-6_2_/IL-6R_2_/gp130_2_) complex or whether both forms can occur [30–33]. Recent evidence suggested that the stoichiometry of the IL-6 signaling complex might be different in classic signaling and trans-signaling [34]. This effect was, however, only described for the murine and not the human receptor complex. For IL-11, only a hexameric signaling complex has been described [35]. Considering that IL-6R and the IL-11R show the same modular organization that allows partial exchange between both receptors without losing their signaling capacity [36, 37], it is tempting to speculate that also the stoichiometry of the signaling complexes might be the same.

Importantly, IL-6R and IL-11R are only expressed on certain cell types, which restricts the number of cells that can be activated by classic signaling [7, 11, 20]. In contrast to that, the signal-transducing receptor gp130 is expressed on all cells which means that IL-6 and IL-11 can act in principle on all cells via trans-signaling. Interestingly, also soluble forms of gp130 exist, which act as natural inhibitors of trans-signaling [38]. For IL-6, it has been shown that classic signaling has rather anti-inflammatory properties, for example, via induction of the synthesis of acute-phase proteins in hepatocytes to combat bacterial infections [39], or as an important factor to induce liver regeneration [40]. In contrast, trans-signaling is primarily regarded as proinflammatory, being critically involved in numerous inflammatory human diseases including inflammatory bowel diseases [41] and atherosclerosis [42]. Accordingly, blockade of trans-signaling was shown to be superior to total blockade of IL-6 signaling, for example, in mouse models for bacterial infection [6, 43, 44]. IL-11 has been initially described to prevent apoptosis and promote platelet maturation [45, 46]. Recently, overshooting IL-11 activity has been associated with the development of epithelial cancers [8, 12, 47]. Studies concerning distinct roles for IL-11 classic signaling or trans-signaling have not been conducted yet.

Soluble cytokine receptors can be generated by two different mechanisms: alternative splicing of the mRNA or proteolytic processing of the membrane-bound receptor. While both mechanisms have been described for the sIL-6R [20, 29, 48], the origin of the sIL-11R is not yet known.

## 3. Generation of the sIL-6R by ADAM Proteases

**Figure 2:**
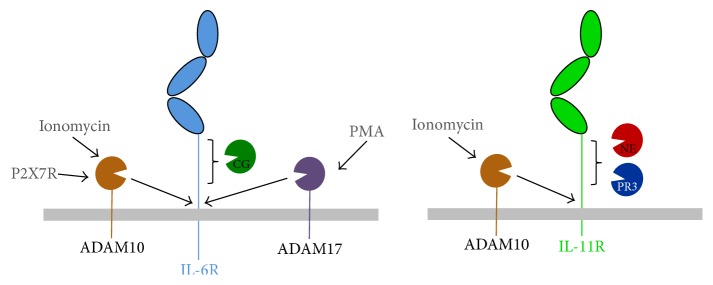
Proteolytic cleavage of the IL-6R and the IL-11R. The IL-6R is a substrate for ADAM10 (activated by ionomycin or via stimulation of the P2X7R), ADAM17 (activated, e.g., via the phorbol ester PMA), or the NSP cathepsin G (CG). The cleavage sites of ADAM10 and ADAM17 are located close to the plasma membrane. CG appears to cleave further upstream, but the exact cleavage site is not known. In contrast, the IL-11R can be cleaved by ADAM10 and the two NSPs neutrophil elastase (NE) and proteinase 3 (PR3). The exact cleavage sites have not been determined so far.

Proteolysis of the membrane-bound IL-6R, resulting in an agonistic soluble IL-6R (sIL-6R), has been demonstrated more than 20 years ago [49, 50]. These initial experiments identified a PKC-dependent mechanism that did not require de novo synthesis of proteins and could be induced by the phorbol ester PMA. Although the responsible protease was not known at that time, inhibitor experiments revealed that it must be a metalloprotease [51], which was subsequently identified as ADAM17 [52, 53] (Figure 2). ADAM proteases are not described in detail in this review, but information can be found elsewhere [54–57].

In an attempt to identify the cleavage site within the IL-6R that is used by ADAM17, COS7 cells overexpressing IL-6R were treated with PMA and the sIL-6R purified from the cell supernatant [58]. Carboxypeptidase treatment of the sIL-6R and analysis of the released amino acids revealed cleavage within the IL-6R stalk region between Gln-357 and Asp-358 [58]. Indeed, a deletion variant of the IL-6R lacking ten amino acid residues from Ser-353 to Val-362 was not shed after PMA treatment [15, 58]. In contrast, a recent paper reported cleavage of a peptide comprising parts of the IL-6R stalk by the recombinant catalytic domain of ADAM17 two amino acid residues further upstream between Pro-355 and Val-356 [59]. Indeed, comparison of known ADAM17 cleavage sites in the MEROPS database [60] makes a cleavage between Pro and Val much more likely, and the IL-6R is the only reported substrate with a cleavage site between Gln and Asp. This is further corroborated by the fact that cleavage site profiling of ADAM17 with the help of peptide libraries revealed a strong preference for a valine residue at the P1′ position as well as a preference for alanine or proline residues at the P1 position [61]. Furthermore, we have recently shown that modelling of the catalytic domain of ADAM17 with the IL-6R stalk peptide also favors cleavage between Pro-355 and Val-356 but not between Gln-357 and Asp-358 [62]. Conclusive data of the exact C-terminus of the sIL-6R generated* in vitro* or even* in vivo*, obtained, for example, via mass spectrometry, are, however, still missing.

A single nucleotide polymorphism within the* IL6R* gene (rs2228145), which causes the insertion of an alanine instead of an aspartic acid residue at position 358, is causative for significantly increased sIL-6R serum levels in healthy humans [63]. This is accompanied by increased serum levels of IL-6 but not sgp130 [64]. Two large-scale analyses incorporating data from more than 100,000 individuals have consistently shown that this SNP is associated with reduced C-reactive protein and decreased odds to suffer from coronary heart disease [65, 66]. Cells from individuals homozygous for rs2228145 show decreased levels of membrane-bound IL-6R [67], and they secrete increased amounts of the differentially spliced sIL-6R isoform [68]. However, this isoform only accounts for a minor proportion of the total amount of sIL-6R [69–72], suggesting that proteolytic cleavage is the major molecular mechanism that generates the sIL-6R* in vivo*. We have shown that the exchange Asp358Ala adjacent to the cleavage site alters the susceptibility of the IL-6R towards proteolysis by ADAMs, making the IL-6R a better protease substrate which is cleaved more efficiently [72]. This appears to be the molecular mechanism which explains the increased serum levels of individuals homozygous for the SNP.

Interestingly, the mechanisms that regulate cellular IL-6R expression are largely unexplored (reviewed in [73]). However, stimulation of cells with the synthetic glucocorticoid dexamethasone induces IL-6R expression [74]. We have further shown that the kinase mTOR plays a central role in modulating IL-6R levels, and activation of mTOR, for example, via EGF signaling, enhances IL-6R expression and sIL-6R generation via proteolysis [75]. Consequently, heterozygous PTEN knock-out mice have increased sIL-6R serum level, highlighting the importance of this pathway* in vivo* [75].

The IL-6R can also be cleaved by ADAM10 [20] (Figure 2). Initially regarded as the protease that is only responsible for the unstimulated, constitutive release of the sIL-6R, several stimuli have been shown to induce IL-6R shedding by ADAM10, including cholesterol depletion [76] and activation of the purinergic P2X7 receptor [77]. Interestingly, the ionophore ionomycin, which increases intracellular calcium concentrations, also induces IL-6R shedding [78], which later turned out to activate ADAM10 [13, 15, 72, 77]. It is currently unknown whether ADAM10 and ADAM17 use the same cleavage site [15] and which of the two proteases contributes to sIL-6R generation in humans. Activated CD4+ T cells have been shown to shed IL-6R mainly by ADAM17 [79]. Physiological activators of IL-6R shedding are rather unexplored but include C-reactive protein [80], ATP [77], IL-1*β*, and TNF*α* [81].

The situation appears to be even more complex in the mouse. Because differential mRNA splicing has been ruled out to contribute to sIL-6R generation [82], proteolytic cleavage of the membrane-bound IL-6R has been suggested as the major mechanism [77, 82]. However, the responsible protease for the steady-state sIL-6R serum levels has not been identified yet. sIL-6R serum levels have been analyzed from mice deficient for either ADAM17 [77], ADAM10 on myeloid cells, ADAM8, or dipeptidyl peptidase I (DPPI) [82], but no reduction has been observed. Although experiments with murine fibroblasts overexpressing IL-6R suggested a species-specific difference in IL-6R proteolysis [77], later experiments with murine cells that express the IL-6R endogenously revealed that ADAM17 is able to cleave the IL-6R [82]. However, in contrast to other substrates like CD62L, shedding of the IL-6R by ADAM17 on T cells appears to be rather weak [77, 83]. In a murine model of LPS-induced acute pulmonary inflammation, mice with a genetic deletion of ADAM17 in leukocytes displayed only 25% reduction of sIL-6R levels in alveolar fluid, suggesting that ADAM17 is not the primary sheddase of the IL-6R [84]. In contrast, the increase in sIL-6R levels one hour after intravenous LPS injection, a model of endotoxemia, was clearly dependent on ADAM17 [83]. Thus, it appears that the mechanisms and/or proteases that control the steady-state serum levels and the inflammation-induced increases in sIL-6R are entirely different.

## 4. Generation of the sIL-11R by ADAM Proteases

The generation of the sIL-11R is not as well studied as the release of the sIL-6R. The existence of sIL-11R in human blood has only recently been described [13] and its origin is far from clear. In mice, transcripts potentially coding for a soluble IL-11R variant have been described [85], but no protein has been detected thus far. In contrast, no mRNA encoding a potential sIL-11R variant has been detected in humans, so that it is still unknown whether a sIL-11R can be generated through alternative splicing.

To analyze proteolytic processing of the membrane-bound IL-11R, ADAM proteases, which have been described to cleave the IL-6R, were considered. Initial attempts focused on the activation of ADAM17 by either LPS or the strong but rather unphysiological stimulator PMA, which have both been shown to induce release of sIL-6R. Interestingly, those stimuli did not induce a change of cell surface amount of either endogenous (macrophages, monocytes) or heterologous expressed IL-11R. Additionally, no sIL-11R could be detected in the supernatant of these cells so that it was concluded that the IL-11R is no substrate for ADAM17 [13, 86]. In contrast to that, activation of ADAM10 via the ionophore ionomycin leads to limited proteolysis of the IL-11R, resulting in loss of endogenous and heterologous cell surface receptor and release of the biologically active soluble ectodomain, which could perform IL-11 trans-signaling* in vitro* [13] (Figure 2). The role of ADAM10 cleavage* in vivo* remains, however, unclear and also whether a sIL-11R is generated in mice is not yet known.

Chimeric IL-6R/IL-11R variants revealed that the protease susceptibility is determined by the stalk region as swapping of that part led to transfer of cleavage specificity [13]. Furthermore, amino acid residue Arg-355 within the IL-11R stalk was shown to be required for efficient ADAM10 mediated proteolysis. According to the cleavage site profiling for ADAM10, arginine residues in P1 or P1′ position are highly favored [61], suggesting that Arg-355 is located at the cleavage site. However, as the cleavage site has not been determined yet, that residue could also be required for IL-11R/ADAM10 interaction without direct involvement in the cleavage event. Besides ADAM10 and ADAM17, other proteolytically active ADAMs exist in humans. Whether these are able to cleave the IL-11R has not been analyzed yet.

## 5. Generation of sIL-6R and sIL-11R by NSPs

Neutrophils belong to the first leucocytes at the site of infection and are a crucial part of innate immunity. Neutrophil-derived serine proteases (NSPs) are a group of highly homologous enzymes found in the azurophilic granules of neutrophils. Their activity is tightly controlled as they are produced as inactive zymogens and require two N-terminal processing steps to gain complete function (removal of signal peptide and further processing by DPPI) [87]. They are stored as fully active proteases and are released from their intracellular pools by activated neutrophils. To date, there are four described family members: cathepsin G (CG), proteinase 3 (PR3), neutrophil elastase (NE), and neutrophil serine protease 4 (NSP4). NSPs possess important functions for the modulation of immune responses. They are involved in the proteolysis of not only several soluble and membrane-anchored substrates such as virulence factors but also chemokines, cytokines, or adhesion molecules [88].

In a first report, it was shown that IL-6R can be shed from the surface of fMLP-activated PMNs and that the sIL-6R was able to activate endothelial cells through trans-signaling. However, the exact mechanism behind the generation of the soluble receptor remained speculative, and a responsible protease was not identified [89]. In addition, Bank et al. demonstrated a positive correlation between the concentration of NSPs and sIL-6R levels in the cerebrospinal fluid of patients with isolated brain injuries. Furthermore, in an* in vitro* setup with pathophysiological relevant concentrations of purified NSPs, they showed that the IL-6R is shed by CG [90] (Figure 2). In accordance with this, McGreal et al. reported degradation of sIL-6R predominantly by CG [91]. Moreover, NSPs contained in the BALF of cystic fibrosis patients were able to cleave sIL-6R, whereas a complex consisting of IL-6/sIL-6R was protected from that degradation [92]. We recently confirmed CG as a sheddase of the IL-6R and additionally also described the IL-11R as a potent substrate for NSPs [13]. In contrast to IL-6R cleavage, a soluble IL-11R fragment was only generated through incubation with purified NE and PR3 (Figure 2). Most importantly both soluble receptors were biologically active and well able to induce cytokine trans-signaling [13].

In conclusion, NSP mediated shedding is considered as potent mechanism for the generation of soluble IL-6R and IL-11R. This could have important implications especially during acute or chronic conditions when high numbers of neutrophils are present.

## 6. Soluble Cytokine Receptors in Human Serum

Agonistic and antagonistic soluble cytokine receptors are found in human body fluids at high concentrations. Serum levels of sIL-6R are usually found at 20–70 ng/mL, and sgp130 serum levels are in the range of 200–400 ng/mL [73]. These serum levels are remarkably stable across individuals, and only a few studies have reported alterations in sgp130 levels during inflammation [73]. In contrast to IL-6, whose concentrations can rise up to 100,000 times during inflammation and infection, sIL-6R serum levels increase only slightly, for example, in rheumatoid arthritis patients [93]. However, a recent paper used serum levels of sIL-6R and sgp130 in combination with IFN*γ* to predict which patients would develop a cytokine release syndrome when treated with chimeric antigen receptor-modified T cells with anti-CD19 specificity against relapsed/refractory acute lymphoblastic leukemia [94]. Nevertheless, the major genetic determinant of sIL-6R levels appears to be the Asp358Ala SNP rs2228145 [63]. Serum levels of sIL-11R are in a much lower range and have only been detected in some healthy humans [13]. Data on sIL-11R in patients have not been published yet.

Interestingly, the biological function of these soluble cytokine receptors in the blood is not known yet. We have recently proposed that sIL-6R and sgp130 together form an IL-6-neutralizing buffer, whose capacity is controlled by the amount of sIL-6R, because sgp130 is present in molar excess [3, 5]. Hereby, IL-6 would bind to the sIL-6R, and the resulting IL-6/sIL-6R complex would be bound and thus neutralized by sgp130, because the IL-6/sIL-6R/sgp130 complex is biologically inactive and is not able to bind to membrane-bound gp130 on target cells. Individuals homozygous for the Asp358Ala SNP would have a higher capacity to buffer and neutralize IL-6, because higher serum levels of sIL-6R are present. This buffer hypothesis might explain why Asp358Ala is associated with a reduced risk to develop coronary heart disease [65, 66]. A similar buffer system can also be envisioned for the sIL-11R in combination with sgp130.

## 7. Specific Inhibition of IL-11 and IL-6 Trans-Signaling by sgp130Fc

**Figure 3:**
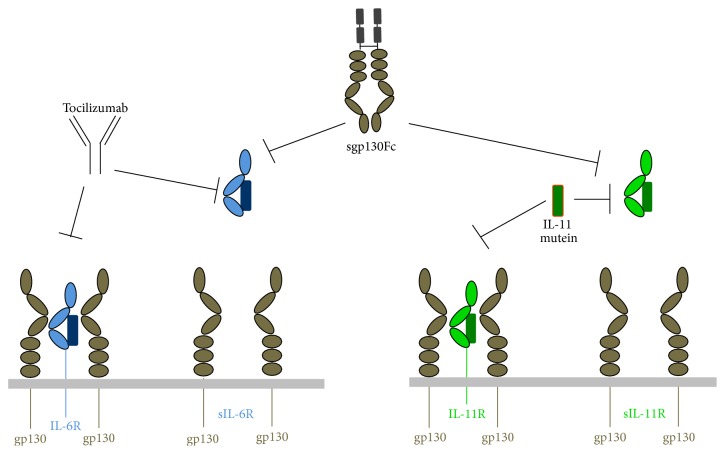
Strategies to inhibit IL-6 and IL-11 signaling. Both sIL-6R/IL-6 and sIL-11R/IL-11 complexes can be specifically blocked by sgp130Fc, which does not bind IL-6 or IL-11 in the absence of their soluble receptors. The monoclonal antibody tocilizumab binds to IL-6R and thus blocks IL-6 classic signaling and trans-signaling. An IL-11 mutein, which binds to the IL-11R but does not activate intracellular signaling via gp130, blocks both IL-11 classic signaling and trans-signaling.

The finding that sgp130 can bind specifically to IL-6 in complex with sIL-6R, but not to IL-6 alone, suggesting that sgp130 is the natural inhibitor of IL-6 trans-signaling [38, 95], led to the development of sgp130Fc, which is a fusion protein of the extracellular part of gp130 with the Fc portion of an IgG antibody [6, 38] (Figure 3). The dimeric sgp130Fc is 10–100 times more potent to inhibit IL-6 trans-signaling than the monomeric sgp130 protein [38, 96]. Under certain* in vitro* conditions, when high amounts of sIL-6R are present, all free molecules of IL-6 can be trapped in IL-6/sIL-6R/sgp130Fc complexes, thus indirectly also affecting IL-6 classic signaling, which can be prevented by using lower amounts of sgp130Fc, allowing classic signaling to happen, while trans-signaling is blocked [96].

Injection of recombinant sgp130Fc or transgenic overexpression of sgp130Fc in mice [97] enabled the analysis of IL-6 trans-signaling in numerous mouse models mimicking human inflammatory diseases (reviewed in [5, 6]). Recently, IL-6 trans-signaling has been shown to be crucially involved in the promotion of Kras-driven lung carcinogenesis [98]. Here, increased sIL-6R levels were detected in the lung of affected mice, and blocking IL-6 trans-signaling with sgp130Fc improved lung cancer pathogenesis [98]. sIL-6R levels were further associated with disease parameters in a murine model of systemic lupus erythematosus, and blockade of the IL-6R improved skin lesions in this model [99].* In vitro*, IL-6 trans-signaling increases the expression of the tumor-associated antigens CEACAM5 and CEACAM6 in colorectal cancer cells, suggesting that sgp130Fc might be a possible therapeutic option in colon cancer [100]. In the kidney, the contributions of IL-6 classic signaling and trans-signaling have been analyzed in detail in two recent studies on glomerulonephritis in mice [101, 102].

IL-11 signaling can be targeted therapeutically via neutralizing antibodies that bind the IL-11R [103] or antagonizing IL-11 muteins, which bind to the IL-11R but do not activate signal transduction via gp130 [104, 105] (Figure 3). One of these muteins has been successfully used* in vivo* [8, 106]. Importantly, neither IL-11 muteins nor neutralizing antibodies are able to discriminate between IL-11 classic signaling and trans-signaling. However, sgp130Fc has recently been shown to efficiently block IL-11 trans-signaling* in vitro* [13]. Although the affinity of IL-11/sIL-11R appears to be lower towards gp130 compared to IL-6/sIL-6R, sgp130Fc nevertheless blocked IL-11 trans-signaling induced cell proliferation and STAT3 activation in a dose-dependent manner [13]. Although the existence of IL-11 trans-signaling has not been shown in mice, it is possible that at least some of the protective effects of sgp130Fc seen in mouse models are not only due to the blockade of IL-6 trans-signaling but also due to inhibition of IL-11 trans-signaling. Furthermore, it might be possible that at least some of the IL-11-driven diseases could be therapeutically targeted with sgp130Fc. More studies are warranted to fully explore the actions of sgp130Fc* in vivo* in order to dissect the contributions of IL-6 trans-signaling and IL-11 trans-signaling in the individual mouse models.

## 8. Concluding Remarks and Outlook

The finding that the signaling of IL-6 is regulated by membrane-bound and soluble forms of the IL-6R has opened up the possibility to either selectively inhibit the trans-signaling pathway via the sIL-6R or globally block both modes of action. Tocilizumab, a humanized antibody that prevents binding of IL-6 to its receptor, is already approved in more than 100 countries worldwide for the treatment of rheumatoid arthritis [2]. Studies with sgp130Fc, solely blocking IL-6 trans-signaling, hold the promise to successfully block the deleterious activities of IL-6 with less side effects. Of note, sgp130Fc has recently passed phase I clinical trials [107].

Although IL-11 has always been described as only active via its membrane-bound receptor, ADAM10 and the NSPs NE and PR3 are able to release the biologically active, soluble ectodomain of the IL-11R, which binds IL-11 with similar affinity as its membrane-bound counterpart. The resulting proteolysis-derived agonistic IL-11/sIL-11R complex appears to act similarly as the IL-6/sIL-6R complex, which is in line with results obtained with recombinant IL-11/sIL-11R proteins [17]. Further studies will elucidate whether the IL-11 trans-signaling pathway is of the same importance as the IL-6 trans-signaling pathway and whether specific inhibition of this signaling mode can be a suitable strategy to treat inflammatory diseases in humans.
